# Perspectives on Harm Reduction Kit Implementation in Heterogeneous Outpatient Clinics

**DOI:** 10.21203/rs.3.rs-6059606/v1

**Published:** 2026-01-25

**Authors:** Raagini Jawa, Margaret Shang, Austen Markus, Megan Hamm, José G. Luiggi-Hernández, Flor de Abril Cameron, Gary McMurtrie, Olivia Studnicki, Mary Hawk, Devon K. Check, Jessica Merlin, Jane M. Liebschutz

**Affiliations:** University of Pittsburgh School of Medicine; University of Pittsburgh School of Medicine; University of Pittsburgh School of Medicine; University of Pittsburgh School of Medicine; University of Pittsburgh School of Medicine; University of Pittsburgh School of Medicine; University of Pittsburgh School of Medicine; University of Pittsburgh School of Medicine; University of Pittsburgh School of Public Health; Duke University School of Medicine; University of Pittsburgh School of Medicine; University of Pittsburgh School of Medicine

**Keywords:** harm reduction, outpatient clinics, implementation science

## Abstract

**Background:**

The evolving U.S. drug market has fueled a public health crisis with rising drug use-associated morbidity and mortality, revealing a mismatch between current abstinence-based addiction care and the needs of people who use drugs (PWUD) to access evidence-based harm reduction services (HRS). Co-locating HRS into outpatient clinics could reduce mortality and improve clinical outcomes. We investigated barriers and facilitators of HRS implementation through kit distribution at three heterogenous outpatient addiction clinics using pre- and post-implementation focus groups.

**Methods:**

We conducted qualitative description approach via 1-hour virtual focus groups and individual interviews with clinic staff and providers both pre- and post-implementation of kit distribution. Interview guides were based on the Consolidated Framework for Implementation Research to assess anticipated and actual implementation barriers and facilitators. Interviews were analyzed using thematic analysis.

**Results:**

Five providers and six staff participated in pre-implementation data collection. Dominant themes pre-implementation included participant enthusiasm for HRS integration and anticipated barriers of personal knowledge and external stigma against PWUD. Six providers and five staff participated post-implementation. Participants reported few actual barriers, of which external stigma and lack of funding for program sustainability were most prominent.

**Conclusions:**

Implementation of HRS in outpatient addiction clinics was well-received by providers and staff and supported by pre-implementation trainings, site champions, and favorable implementation environments. Further efforts are needed to reduce stigma in the greater community and achieve sustainable funding for HRS.

## Introduction

Rapid and dynamic changes within the U.S. drug market have taken place in the past several years. Potent adulterants with sedating properties like fentanyl and xylazine dominate the unregulated opioid supply, which has contributed to the overdose crisis, in which over 100,000 deaths occurred in 2022 (1). In parallel, rates of infectious complications like hepatitis C, human immunodeficiency virus, and serious bacterial infections have risen at an alarming pace. This has resulted in in increased hospitalizations and higher mortality rates among people who use drugs (PWUD) (2), placing significant financial strain on healthcare systems (3).

Harm reduction is a set of strategies and principles aimed at minimizing health risks associated with drug use without necessitating cessation (4). The most visible harm reduction services (HRS) include syringe service programs (SSPs), naloxone distribution, and overdose prevention hotlines like SafeSpot. The community organizations and mutual aid groups that deliver these services are often siloed from healthcare settings and limited by staff and funding to provide necessary coverage to an exponentially growing population in need (5).

Outpatient clinics offering addiction treatment, including primary care clinics and Federally Qualified Health Centers, are ideal settings for integration of HRS. While historically seen as conflicting with abstinence-based treatment goals, integrating HRS in these settings can promote a patient-centered approach, recognizing that some PWUD may wish to continue using substances in a safer manner or may not be ready to stop (6). Unlike SSPs, which remain illegal in some states, outpatient clinics offer broader geographic coverage, extended hours, and can deliver medical services like addiction treatment, vaccinations, and bloodborne viral infection screenings. Modeling studies have shown that integrating HRS in outpatient clinics is cost-effective. This approach can reduce drug-related mortality by 30%, enhance provider-patient relationships, encourage treatment initiation, and ultimately reduce acute healthcare usage (7),(8). This approach aligns with national efforts, such as the 2021 Biden-Harris National Drug Control Strategy and 2023 Health Resources and Services Administration guidance, to expand HRS access to outpatient settings (9).

Despite growing support for incorporating HRS into outpatient clinics, (10, 11) practical implementation methods remain underdeveloped outside of hospital settings(12, 13). Previous research, albeit limited to isolated clinics, has shown that integrating harm reduction into medical settings is challenging, with embedded stigma towards PWUD, (14) legal and political opposition to compassionate care practices, (15) and prioritization of abstinence-only substance use disorder treatment modalities over other evidence-based treatment strategies (16). Since integrating comprehensive addiction treatment and harm reduction in outpatient clinics can address the needs arising from the evolving drug market, it is critical to explore how HRS can be effectively implemented into these settings. To address this gap, we explored barriers and facilitators to implementing HRS at three office-based addiction treatment programs.

## Methods

This qualitative study was conducted to evaluate an initiative to implement harm reduction kits within office-based addiction treatment settings. We took a qualitative description approach (17, 18) guided by the implementation determinant framework Consolidated Framework for Implementation Research 2.0 (CFIR) (19) and sought to thoroughly and accurately describe participants’ thoughts and feelings on interview topics; this approach is common in qualitative studies in medical and health sciences contexts. We chose to conduct pre- and post-implementation data collection to assess both anticipated and actual barriers and facilitators to implementation to guide future efforts.

### Settings and Participants

Since 2016, the Pennsylvania Department of Human Services has certified over 200 behavioral and physical health entities as Centers of Excellence for Opioid Use Disorder (COEs) including primary care practices, and office-based addiction treatment programs which aim to provide evidence-based treatment for PWUD. Core components of COEs include providing comprehensive case management services and Certified Recovery Specialist support to address social determinants of health and increasing access to medications for opioid use disorder (MOUD) which have demonstrated to increase patient retention (20). This network supports COEs as ideal venues for scaling evidence-based interventions. Our study occurs at three urban COEs with heterogeneous treatment settings in Western PA: a dual-diagnosis clinic, a hospital-based pregnancy and women’s recovery clinic, and a family medicine primary care clinic. All sites expressed interest in implementing harm reduction kits (safer injection, safer smoking, safer snorting, safer boofing (per-rectal administration of drugs), fentanyl and xylazine test strips, and wound care kits) (21) and identified faculty leads and staff champions (site champion hereafter) for their respective COE.

In August 2023, we reached out to each site champion to help recruit for pre-implementation focus groups via their clinic listserv and regularly scheduled clinic meetings. Using purposive sampling to ensure adequate representation from each COE, we targeted recruiting at least two staff members and at least two providers from each COE to participate in separate staff and provider focus groups. Four months after our initiative began in February 2024, we recruited participants for our post-implementation focus groups using the same strategies.

### Data Collection

The pre- and post-implementation focus group interview guide (see Appendix) was created based on CFIR 2.0, which is a widely used determinants framework to explore barriers and facilitators in implementation of interventions. It includes five domains—intervention characteristics, outer setting, inner setting, individual characteristics, and process—with 26 constructs in total. Questions included current experiences with HRS, anticipated (pre-implementation) and actual (post-implementation) barriers and facilitators to implementing harm reduction kits within each clinic, and anticipated workflow for integration.

In September 2023, an experienced qualitative methodologist (MH) conducted two pre-implementation hour-long focus groups via Zoom. Given challenges associated with recruiting busy front-line providers, our approach was flexible and allowed for the option of one-on-one interviews and focus groups with fewer than 5 participants, which are regarded as group interviews. Participants provided verbal consent prior to focus groups/group interviews. In May-June 2024, the same methodologist (MH) conducted two post-implementation focus groups via Zoom. Due to a scheduling conflict, two providers were interviewed together. Participants were compensated $25 for the pre-implementation and $30 for the post-implementation focus groups.

### Data Analysis

All focus groups and interviews were audio-recorded, transcribed verbatim, and de-identified. A codebook was developed both deductively based on the CFIR 2.0 framework and inductively based on recurring topics identified. The codebook was then applied to all transcripts by two experienced coders (MH and FC), and the primary coder (MH) compared and adjudicated all coding to ensure that codes were applied consistently. The primary coder then used the completed coding to conduct first a conventional content analysis, (22) and secondarily a thematic analysis (23, 24) based on both coding and discussion of content analysis with the rest of the study team. This study was approved by the University of Pittsburgh institutional review board.

## Results

A total of 5 providers (1 interviewed individually, 4 in a group interview) and 6 non-clinical staff participated in pre-implementation data collection (see Table 1). Post-implementation, a total of 6 providers (2 in a group interview, 4 in a group interview) and 5 staff participated.

### 1. Pre-implementation, participants were enthusiastic about providing HRS in their clinics.

Participants entered implementation feeling receptive to providing HRS and felt that their staff, providers, and patients would be enthusiastic. Some clinics were already distributing naloxone and/or drug checking supplies and noted that doing so had been a positive experience:

I think it really helps to create therapeutic relationships with patients when they know that you’re sort of honoring their safety first and foremost. I feel like people feel… the care we’re giving is a lot less judgmental. And through conversations about harm reduction, I find that patients feel like they’re able to open up more.

Another provider noted that she felt “*more comfortable sending our clients back out into the community after the visits, knowing that they may be a little safer if they do end up using again.*” Overwhelmingly, provision or expansion of HRS was in alignment with clinic goals and federal priorities, and the anticipated within-clinic reception was almost universally positive with multiple participants describing themselves as “*excited*.”

Harm reduction was viewed as a valuable service for patients and their communities. One participant described:

I think the more harm reduction we can offer our community, not just our patients, the better we all are as a community. I like to make the parallel with education: the more educated a population is, the better everybody is going to do regardless of how educated or not educated the specific person is. So, the more harm reduction we can put into our communities, the better every person in our community will do, whether they want it, need it, seek it out, or they don’t.

While participants anticipated needing time to work out the logistics of kit distribution (i.e., clinic-specific workflows, integration of kits into physical and work infrastructure like kit storage or staffing, inventory management, etc.), those issues were not viewed as insurmountable:

I don’t see it being much of an increase or a burden. I feel like we’re already educating on some, many of these things, we just don’t have the physical supplies… it’s gonna be such a great tool for us to actually have them there, so we can use them while we’re educating, and I don’t think it’s going to take much more time or any, or anything like that. We’re already talking about most of it.

### 2. Pre-implementation, participants identified anticipated barriers related to clinicians’ self-efficacy and external stigma against PWUD.

When describing barriers, participants noted a need for harm reduction training on safer use strategies and use of some supplies in the kits, particularly for less-familiar drug use methods like boofing (per rectal administration) and snorting.

Additionally, providers at some COEs acknowledged potential barriers external to the clinic, most notably public and healthcare-related stigma and a focus on abstinence-based care in the broader health system(s). This was highlighted by one participant working in a hospital-based clinic:

I think stigma is a huge [barrier]. At the women’s hospital in particular, especially when you’re talking about women who are pregnant or potentially becoming pregnant, I think this idea is not well-received by the community at large. That it’s still seen, very unfortunately, as sort of enabling patients to have ongoing use. We see that a lot in the work that we do, unfortunately.

Another provider from this clinic described that previously, hospital pharmacy leadership had prevented them from distributing naloxone at an Overdose Awareness event. Participants from another COE noted that the clinic had previously distributed sterile syringes but were stopped due to senior leadership changes. Participants also noted that patient encounters with law enforcement could be negative if found in possession of drug use equipment:

I know at the needle exchange, people are given cards that say like, I’m a member of [Name of Needle Exchange Program], and they’re told, if you have any sort of negative interaction with law enforcement, you can show them this to say this is where you obtained this equipment. I don’t know how common..., even though it’s legal for them to be receiving these supplies, that… it actually does cause a problem for them… Speaking of harm reduction, I would certainly hate to be part of something that actually caused harm or caused problems for people.

They also noted lack of clarity on local laws and institutional policies surrounding HRS. Potential legal issues were described as particularly pronounced for anyone with a history of incarceration, living in mandated abstinence-based recovery housing, and/or with Child Protective Services (CPS, also referred to as CYF) involvement. Even without legal concerns, some providers mentioned the importance of having conversations with patients upfront to ensure a secure place to store kits to mitigate potential conflicts or stigmatizing interactions with family, partners, and/or roommates.

#### Implementation Process:

All sites received pre-implementation training on the evidence behind harm reduction and site champions helped develop COE-specific workflows.(21) Most clinics settled on a model in which patient-facing menus listing kit options were universally provided upon check-in and reviewed by staff or providers who then gave selected kits to patients. Personnel considerations were made to ensure that certain staff such as Certified Recovery Specialists were not placed in positions in the workflow that could compromise or jeopardize their recovery.

### 3. Post-implementation, participants described an experience with few actual barriers, and any barriers were largely associated with external stigma.

Participants primarily felt that implementation of kit distribution was successful, noting few actual barriers. One clinic noted higher traffic in the room storing kits and had to navigate ensuring the materials were routinely accessible. COEs embedded within larger practices treating other patient populations reported additional barriers that prevented completely integrating kit distribution within their clinic workflow. Examples included having to omit kit-specific messaging in waiting rooms shared with pediatric patients and rely on clinical staff or providers to identify and provide menus to the appropriate patients in lieu of universal distribution of menus upon check-in. Participants noted that kit implementation was positively received by patients, who felt that the presence of harm reduction kits in the clinic indicated a broader concern for their health and a desire to continue a therapeutic alliance despite return to use, directly opposing punitive abstinence-based practices. As one person described:

I think the other piece, too, that’s been really great with harm reduction is, again, just for patients that, are inconsistent with their bup[renorphine] use, or have, returned to use, instead of, discharging them from the clinic, or saying, hey, let’s take a break and have the social worker reach out to you in a month, we’re able to just still see them and offer harm reduction, instead. And so, that’s been pivotal at keeping people engaged.

Difficulties arising from kit distribution largely reflected stigma at various levels: the individual level through internalized stigma and fear of disclosure of active use among patients, health system level, and the community-level through issues with CYF, law enforcement, and pharmacies. These issues were rare, but in some cases carried the potential of serious negative impacts on clients. For example, one clinic reported having to write a letter on behalf of a patient regarding a single incident in which a CYF worker raised concerns about their possession of the kits:

[We had one] patient that was searched, that had the testing strips and was being pushed by CYF about paraphernalia… that was the only bump that I’ve really had with it… I told the CYF worker, ‘it’s all labeled’. And the CYF worker was like, ‘Anyone can print out stickers. There’s no doctor’s name signed to that.’ So that’s why the onus of needing to have a letter [had] to happen… CYF will always have a reaction... So that’s certainly a bump, and it only happened one time.

Another clinic described an incident with a community pharmacy, which taught them the importance of educating patients upfront on potential risks and/or negative interactions to allow for informed decision making and explicitly developing partnerships with local pharmacies:

Our provider had written a prescription for syringes, and it was to more of a rural pharmacy, and the staff at that pharmacy was very judgmental. And even said, if you pick these up, we could notify the police that you’re getting syringes and you don’t have a medical disorder like diabetes or something that would be required to do it, so the patient just kind of left at that point. So, it helped us… be able to be like, okay, well, we’ll fill at our pharmacy here, and you can take them with you, like that type of situation, trying to mitigate those risks for the patient, and again, being upfront and honest with them about it, I think, was a key component… making sure that they were understanding of the risks.

### 4. Post implementation, lack of funding was the primary barrier to sustainability.

Participants expressed concerns for ensuring adequate funding for supplies and labor to assemble and distribute the kits following the study period, particularly since HRS are not considered billable services with one participant noting:

At our clinic, we would need some time [to figure out sustainability], because we’ve benefited from the study [team] taking care of that [kit packing]. The study has taken care of the ordering. The study has taken care of the kit packing and the delivery to our locations. So, we would need to figure out logistically some of those things, especially concerning who’s going to pack the kits? How are we going to compensate them? That sort of thing.

Some clinics anticipated using internal funds for these purposes, but others were actively seeking external grant funding opportunities. Despite this uncertainty, participants universally wanted to continue kit distribution and even desired to expand this initiative to other settings within their health systems. Participants found that there was ideological support for continued kit distribution and that HRS provision aligned well with clinic culture and goals:

So, for us, I have been speaking with our medical director who would like to… expand this to the other clinics. This is where we’re, again, like a bigger swing, so with that, I think there’s a lot of support. Unfortunately, it just kind of goes back down to, like, the funding of where is it going to come from… But I think we’ve received a lot of support. It’s more just… how do we make it work?

## Discussion

We found that implementation of office-based HRS in heterogenous COEs was desirable among staff and providers and aligned well with clinic priorities. While there were several anticipated barriers to kit distribution, many were addressed through implementation strategies such as site champions and tailored site-specific workflows. Remaining or unaddressed barriers including substance use stigma from government agencies and community pharmacies at the outer setting level, underscoring the challenge of HRS implementation in light of societal attitudes that prioritize abstinence and criminalization over treatment. Consistent with prior literature, such pervasive societal and healthcare stigma against PWUD embedded in federal and state laws and health care institutional policies serves as a compelling barrier to effective implementation of office-based HRS (25). Despite this, we found that advocacy at the individual and inner setting levels helped to combat some of these obstacles. Site champions were instrumental as change agents within the broader inner setting’s ecosystem (26, 27) through educating their colleagues and streamlining kit distribution at their respective sites. Moreover, providers and staff took initiative to intervene on behalf of patients encountering stigmatizing interactions, highlighting the importance of advocacy and education in addressing healthcare-related stigma within healthcare settings. Future efforts should proactively include community partners throughout implementation and contingency plans for legal challenges to protect patients, providers, and staff (28).

While COEs are well-positioned to adopt HRS as a part of the addiction care continuum,(29) we found that inadequate self-efficacy of harm reduction practices served as a barrier to implementation, which we addressed through pre-implementation trainings and site champions. This aligns with prior evidence demonstrating a significant curricular gap in health sciences education which often focuses on abstinence-based measures and medications over harm reduction (30–33). While isolated educational trainings on naloxone and safer use have led to increased comfort and adoption among health professionals, (34–36) future efforts should be made to integrate these trainings early in medical education where there is growing attention in developing addiction medicine curricula and within mandated trainings such as the one-time 8-hour education requirement for all Drug Enforcement Administration prescribers. Other potential sources of harm reduction education include asynchronous trainings, local training and technical assistance programs, learning collaboratives, and partnerships with harm reduction organizations and persons with lived or living experience.

Most notably, the financial sustainability of implementing office-based HRS remained a concern both pre- and post-implementation among participants and is an unfortunate reflection of the long-standing funding barrier faced by the greater community-based harm reduction organizations (37). Clinics were worried that they would bear the financial burden of sustaining HRS, despite evidence suggesting that it would be cost-effective for health systems by promoting patient engagement in addiction care and reducing substance use-associated acute visits and mortality,.(10) Funding for HRS is critical for program sustainability and scaling into outpatient clinics, yet has not been a proportional governmental investment in harm reduction programming (37). Given this, strategies to cover HRS under Medicare and Medicaid should be explored, emulating advancements made in expanding coverage for addiction treatment, patient navigation services, and contingency management (38). In the interim, given the affordability of kits, (21) health systems could partner with mutual aid health groups to procure harm reduction materials (39) and consider utilizing available on-site staff for kit assembly and inventory management through task shifting as low-cost strategies (40).

## Conclusion

The implementation of office-based HRS in COEs was generally well-received due to a favorable environment, pre-implementation trainings, site champions, and site-specific workflows. External stigma from abstinence-focused treatment communities and pharmacies remained a barrier but was addressed through advocacy and education from clinic providers and staff. More importantly, financial sustainability for HRS remains a major challenge, and while low-cost strategies like utilizing mutual aid networks and task-shifting can support these services in the interim, advocacy for broader policy change and federal investment in harm reduction programs is needed to facilitate widespread implementation and ensure long-term success.

## Supplementary Material

Supplementary Files

This is a list of supplementary files associated with this preprint. Click to download.

• HRKTable1.docx

• HRKAppendix.docx

## Figures and Tables

**Figure 1:**
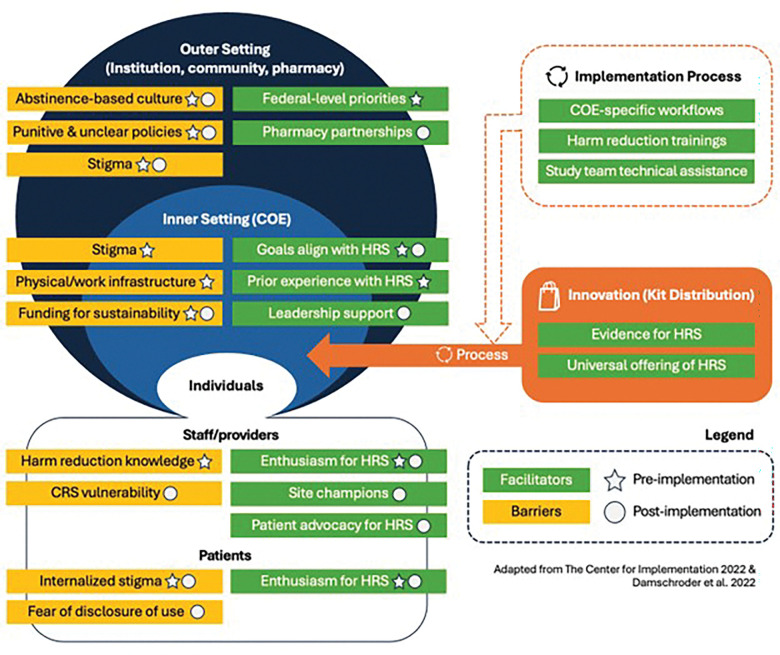
Barriers and facilitators for implementation of office-based HRS mapped on CFIR Abbreviations: CFIR, Consolidated Framework for Implementation Research; COE, Center of Excellence for Opioid Use Disorder; CRS, Certified Recovery Specialist; HRS, harm reduction services

**Table 1:** Staff and provider participants by type in pre- and post-implementation focus groups

	Pre-implementation	Post-implementation

**Providers**		
Physician	2	3
Nurse practitioner	1	1
Clinicians^a^	2	2

**Staff**		
Nurse	1	1
Administrator	2	1
Peer navigator	2	1
Care manager/coordinator	1	2

a Clinicians are defined as licensed clinical social worker. We included clinicians at one of the participating COEs in the provider focus group as they functioned primarily as a behavioral health entity.

## Data Availability

The qualitative data can be made available upon reasonable request to the corresponding author, subject to approval from the institutional review board.

## Highlights

Pre-implementation, clinic providers and staff were eager to integrate harm reduction services (HRS) at their sites, though they anticipated logistical challenges incorporating these into existing busy workflows.

External stigma from health care systems, law enforcement, and public towards people who use drugs remained significant post-implementation barriers; however, education and advocacy from site champions and clinic providers and staff helped mitigate these challenges.

While clinics were keen to continue and expand HRS, a key post-implementation challenge was securing the financial sustainability for the services.

## Abbreviations

COE: Center of Excellence for Opioid Use Disorder
CFIR: Consolidated Framework for Implementation Research
CPS: Child Protective Services
CRS: Certified Recovery Specialist
HRS: harm reduction services
PWUD: people who use drugs
SSP: syringe service program
